# Case Report of Schnyder Corneal Dystrophy—A Rare Lipid Metabolic Disorder of the Cornea

**DOI:** 10.3390/life15030409

**Published:** 2025-03-06

**Authors:** Nina Stoyanova, Abdulrahman Imran, Zain Ul Hassan, Krasimir Kraev, Yordanka Basheva-Kraeva, Maria Kraeva, Petar Uchikov, Plamena Novakova, Veselin Vasilev, Ivaylo Minev, Bozhidar Hristov, Desislava Koleva-Georgieva, Petko Petrov, Luboslav Dimov, Svetlan Dermendzhiev, Marin Atanassov

**Affiliations:** 1 Department of Ophthalmology, Faculty of Medicine, Medical University of Plovdiv, 4002 Plovdiv, Bulgaria; nina.st.st@abv.bg (N.S.); dannybasheva@gmail.com (Y.B.-K.); desislava.koleva@mu-plovdiv.bg (D.K.-G.); marin_aa@abv.bg (M.A.); 2 Faculty of Medicine, Medical University of Plovdiv, 4000 Plovdiv, Bulgaria; imran.ar057@gmail.com (A.I.); zulhassan2002@gmail.com (Z.U.H.); 3 Department of Propedeutics of Internal Diseases, Medical Faculty, Medical University of Plovdiv, 4002 Plovdiv, Bulgaria; 4 Department of Otorhinolaryngology, Medical Faculty, Medical University of Plovdiv, 4002 Plovdiv, Bulgaria; kraevamaria93@gmail.com; 5 Department of Special Surgery, Medical Faculty, Medical University of Plovdiv, 4002 Plovdiv, Bulgaria; puchikov@yahoo.com; 6 Department of Allergy, Medical Faculty, Medical University of Sofia, 1000 Sofia, Bulgaria; nplamena@yahoo.com; 7 Department of Physiology, Medical Faculty, Medical University of Plovdiv, 4002 Plovdiv, Bulgaria; veselin.vasilev@mu-plovdiv.bg; 8 Department of Anaesthesiology, Emergency and Intensive Care Medicine, Medical Faculty, Medical University of Plovdiv, 4000 Plovdiv, Bulgaria; ivaylo.minev@mu-plovdiv.bg; 9 Second Department of Internal Diseases, Section “Gastroenterology”, Medical Faculty, Medical University of Plovdiv, 4002 Plovdiv, Bulgaria; hristov.bozhidar@abv.bg; 10 Department of Maxillofacial Surgery, Faculty of Dental Medicine, Medical University of Plovdiv, 4000 Plovdiv, Bulgaria; petkogpetrov0@gmail.com; 11 Department of Endocrinology, Medical Faculty, Medical University of Plovdiv, 4000 Plovdiv, Bulgaria; lyuboslav.dimov@phd.mu-plovdiv.bg; 12 Department of Occupational Diseases and Toxicology, Medical Faculty, Medical University of Plovdiv, 4000 Plovdiv, Bulgaria; svetlan_d@yahoo.com

**Keywords:** Schnyder corneal dystrophy, lipid metabolism, tocilizumab-induced dyslipidemia

## Abstract

Background: Schnyder corneal dystrophy (SCD) is a rare autosomal dominant disorder characterized by bilateral corneal opacification due to abnormal cholesterol and phospholipid deposition. Mutations in the UBIAD1 gene, identified as causative in 2007, underline the condition, although its exact pathogenesis remains unclear. Case Presentation: A 55-year-old female presented with persistent photophobia, blepharospasm, and corneal discomfort. She also reported joint pain related to rheumatoid arthritis (RA), managed with Ro-Actemra (tocilizumab). The ophthalmological evaluation revealed bilateral corneal stromal deposits resembling snowflakes, with visual acuities of 0.8 (right eye) and 0.7 (left eye). Multimodal imaging confirmed stromal hyperreflective deposits. Based on the clinical findings, SCD was diagnosed, although no genetic testing was performed. Symptomatic management with artificial tears was initiated. Discussion: This case illustrates the diagnostic challenges of SCD, particularly in the absence of corneal crystals, a hallmark feature that is not universally present. Advanced imaging techniques aided diagnosis, and the coexistence of SCD and RA highlights the need for multidisciplinary care. Treatment options remain limited, although emerging therapies targeting oxidative stress and lipid metabolism show promise. Conclusions: This case highlights the importance of integrating ophthalmological and systemic care in SCD management and underscores the need for further research to expand diagnostic and therapeutic strategies for this rare disorder.

## 1. Introduction

**Figure 1:**
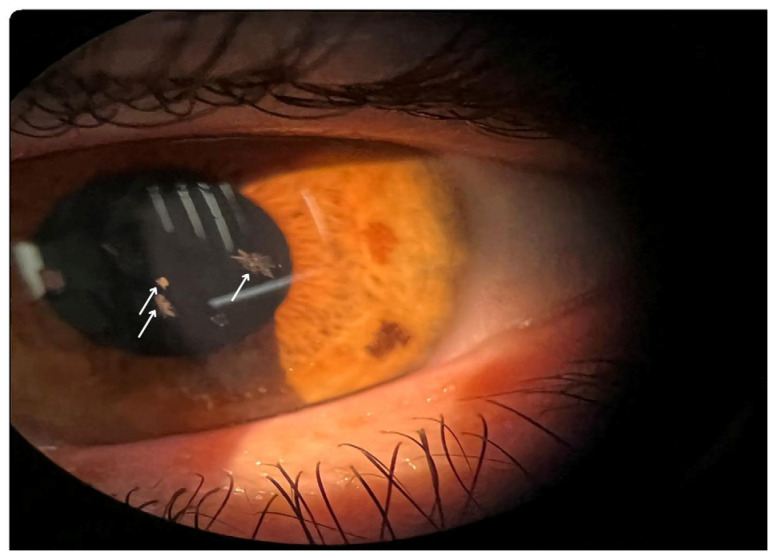
Crystalline corneal deposits in SCD, reflecting abnormal lipid accumulation and disease progression.

Schnyder corneal dystrophy (SCD) is a rare, progressive autosomal dominant disorder that was first described by the Swiss ophthalmologist Franz Schnyder in 1924 [1,2,3]. It has a prevalence estimated at less than 1 in 1,000,000 individuals. It is characterized by bilateral opacification of the cornea due to abnormal accumulation of lipids, primarily cholesterol and phospholipids, within the corneal stroma (Figure 1) [4]. This lipid deposition leads to progressive corneal clouding and visual impairment, often beginning in early adulthood and advancing with age [5]. Clinically, SCD is notable for its variable presentation, ranging from subtle stromal haze to conspicuous crystalline deposits, depending on the stage and severity of the disease [6,7].

The genetic basis of SCD was elucidated in 2007, with the identification of mutations in the UBIAD1 gene as the primary causative factor. UBIAD1 encodes a key enzyme that is involved in cholesterol metabolism and vitamin K2 biosynthesis, underscoring the metabolic underpinnings of the disease [8]. However, the exact molecular mechanisms linking UBIAD1 mutations to corneal lipid accumulation remain poorly understood. Dysregulated lipid metabolism is thought to impair corneal transparency, leading to progressive visual decline [9,10,11].

While traditionally considered a corneal disorder, SCD is increasingly recognized for its potential systemic implications, including dyslipidemia. Advances in imaging modalities, such as optical coherence tomography (OCT) and confocal microscopy, have enhanced the diagnostic accuracy, particularly in cases lacking visible corneal crystals. Despite these advancements, treatment options remain limited, with penetrating keratoplasty being reserved for advanced cases [11,12,13,14,15].

This case underscores the importance of recognizing rare corneal dystrophies such as SCD, particularly in patients with systemic comorbidities or atypical corneal findings. Enhanced clinical awareness and improved diagnostic strategies could facilitate earlier diagnosis, timely management, and better visual outcomes for affected individuals.

## 2. Case Presentation

### 2.1. Patient History

A 55-year-old female presented with persistent ophthalmic complaints, including pain and photophobia, necessitating the frequent use of sunglasses. She also reported joint pain in the hands, specifically involving the metacarpophalangeal (MCP), proximal interphalangeal (PIP), and distal interphalangeal (DIP) joints, as well as in the right knee. Her medical history was significant for rheumatoid arthritis, managed with RoActemra (tocilizumab).

### 2.2. Clinical Findings

**Figure 2:**
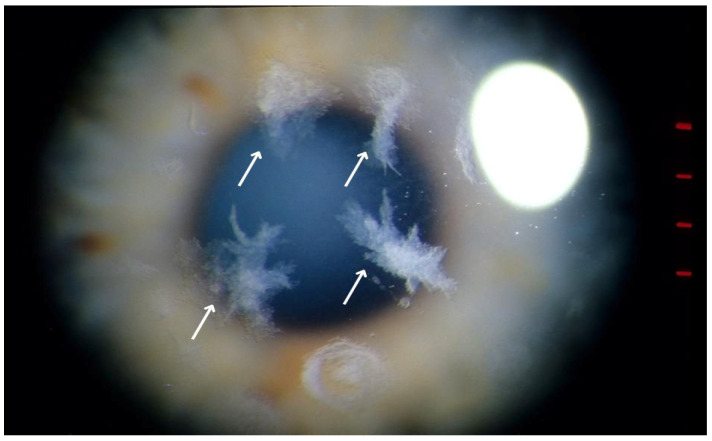
Bilateral corneal stromal deposits with a snowflake-like pattern, characteristic of Schnyder corneal dystrophy (SCD), resulting from abnormal lipid accumulation and contributing to corneal clouding and visual impairment.

An ophthalmological examination revealed bilateral corneal stromal deposits resembling snowflakes (Figure 2), a characteristic finding of Schnyder corneal dystrophy (SCD). The visual acuity of the right eye was 0.8 (VOD), with an intraocular pressure of 17.5 mmHg (TOD). The left eye had a visual acuity of 0.7/0.8 (VOS) and an intraocular pressure of 14.1 mmHg (TOS). Both eyes exhibited the following:

- Blepharospasm;
- A calm conjunctiva;
- A clear and normally deep anterior segment;
- A round and centered pupil that reacted to light;
- A transparent lens.

### 2.3. Diagnostic Assessment

The diagnosis of Schnyder corneal dystrophy was established based on the clinical findings of bilateral corneal stromal deposits and the patient’s ophthalmic symptoms. No genetic testing was performed, but the characteristic corneal features supported the diagnosis. Additionally, laboratory tests revealed elevated serum cholesterol (7.1 mmol/L) and triglyceride levels (4.8 mmol/L), further supporting the metabolic nature of the disease.

### 2.4. Therapeutic Interventions

The patient was managed with artificial tears for symptomatic relief of her ocular discomfort and photophobia. Her ongoing treatment with Ro-Actemra for rheumatoid arthritis was continued to manage systemic symptoms. A multidisciplinary approach was emphasized to address the coexistence of SCD and rheumatoid arthritis.

## 3. Discussion

### 3.1. Clinical Presentation and Diagnostic Challenges

The clinical presentation of SCD has evolved in its understanding over time. Weiss et al. [1] conducted a cohort study of 115 patients and revealed that corneal crystals, long considered a hallmark of SCD, are present in only 54% of cases. This finding challenges traditional diagnostic perceptions, underscoring the need to consider SCD even in the absence of visible crystals.

**Figure 3:**
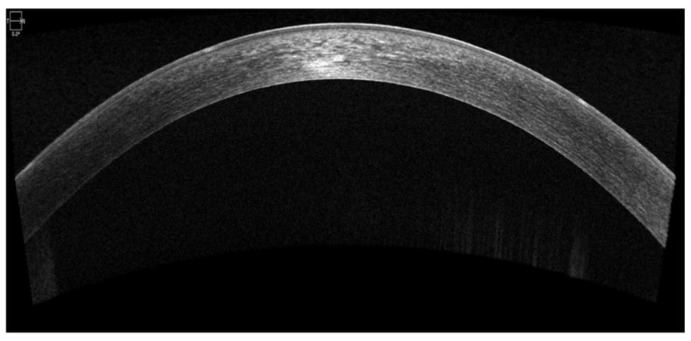
Optical coherence tomography (OCT) image of the cornea, demonstrating stromal hyperreflective deposits. The corneal architecture remains intact, with no evidence of significant thinning or scarring.

**Figure 4:**
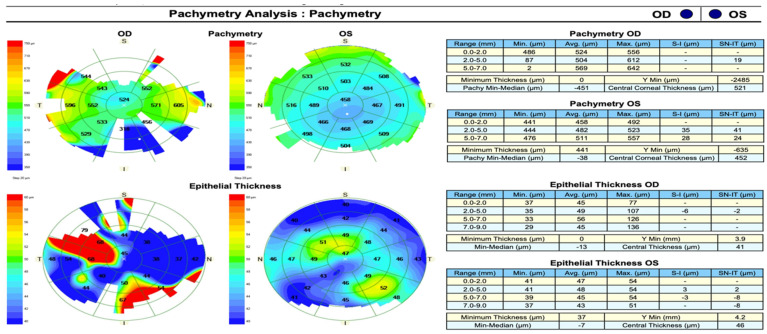
Pachymetry and epithelial thickness maps of both eyes. The right eye (OD) exhibits focal epithelial thickening, while the left eye (OS) demonstrates mild central thinning, consistent with lipid deposition affecting corneal structure.

This case illustrates the diagnostic complexity of Schnyder corneal dystrophy (SCD), particularly in the absence of visible corneal crystals. While the characteristic snowflake-like stromal deposits that were observed in this patient aligned with descriptions of SCD in advanced stages, corneal crystals—a traditionally defining feature—were not universally present, aligning with Weiss et al.’s findings. The patient’s additional symptoms, such as photophobia and blepharospasm, underscore the broader impact of SCD on patients’ quality of life, beyond vision impairment. These findings emphasize the need for a comprehensive clinical evaluation and the importance of recognizing atypical presentations. In atypical cases, additional tools must be employed to aid in diagnosis, such as OCT imaging and pachymetry (Figure 3 and Figure 4).

Making a diagnosis can be challenging in SCD due to the variability in presentation and incomplete penetrance of crystallization in the cornea. The early presentation can be in the form of corneal haze without specificity and can be mistaken for corneal dystrophies or degeneration. The lack of family history in some cases and overlap with lipid corneal deposition in systemic dyslipidemia can be complicating factors. The use of a slit-lamp alone may be insufficient for identifying mild stromal abnormalities, and hence, supportive imaging tools like OCT and confocal microscopy are required.

### 3.2. Differential Diagnoses

The differential diagnoses of corneal lipid deposition must include a range of conditions beyond SCD. These include systemic lipid metabolism disorders such as hyperlipidemia, corneal arcus secondary to dyslipidemia, and certain lysosomal storage diseases like Fabry disease. A key consideration in this case was the potential impact of the patient’s ongoing treatment with Ro-Actemra (tocilizumab) for rheumatoid arthritis. Tocilizumab, an IL-6 receptor antagonist, has been associated with alterations in lipid profiles, including elevated cholesterol and triglyceride levels. While the exact contribution of Ro-Actemra to the corneal findings is unclear, it raises the possibility that lipid dysregulation induced by the medication may have exacerbated or contributed to the lipid accumulation in this patient’s cornea. In this case, a serum lipid assessment confirmed elevated cholesterol and triglyceride levels, reinforcing the metabolic component of SCD. This finding further supports the need for regular lipid monitoring in patients with SCD, particularly those receiving IL-6 inhibitors such as tocilizumab, which has been linked to dyslipidemia. This underscores the need for a thorough systemic and pharmacological history in patients presenting with atypical corneal findings.

### 3.3. Systemic Implications

The systemic dimensions of SCD remain a critical yet underexplored area. Dyslipidemia, which has a well-documented association with SCD, was not explicitly evaluated in this patient. Kurtul et al. [4] described a 34-year-old male with SCD and dyslipidemia, highlighting the importance of systemic co-management. Similarly, our patient presented with joint pain and was undergoing treatment for rheumatoid arthritis with RoActemra, emphasizing the need for a multidisciplinary approach. While no direct link has been established in the literature, the coexistence of these conditions suggests a need for further investigation into their potential interplay.

### 3.4. Advances in Molecular Understanding

The genetic and molecular underpinnings of SCD, particularly the role of UBIAD1 mutations, have been thoroughly established in the literature. Nowinska et al. [2] identified a novel mutation (I245N) and confirmed a known mutation (N102S) in a Polish cohort. Their use of advanced imaging techniques like optical coherence tomography (OCT) provided detailed morphological insights, similar to the multimodal imaging that was utilized in the diagnosis of our patient. However, the lack of genetic testing in our case represents a limitation, as identifying specific mutations could provide insights into the disease progression and prognosis.

### 3.5. Potential Treatment Approaches and Future Directions

The current treatment landscape for SCD remains limited, with penetrating keratoplasty being the primary option for advanced cases. Weiss et al. [1,11,15,16,17,18] suggested its effectiveness for patients with photopic vision loss who are over 50 years old, but the risk of disease recurrence in the donor cornea remains a concern. In this case, the use of artificial tears provided symptomatic relief, reflecting the constrained options that are available for early or less severe diseases.

Recent research has explored novel therapeutic approaches that target the underlying pathophysiology of SCD:

- Topical Statins: Given the lipid metabolism dysfunction in SCD, statins such as atorvastatin and simvastatin have been investigated for their ability to reduce corneal cholesterol deposition. Preliminary studies suggest that they may slow disease progression by modulating lipid homeostasis.
- Antioxidants and Autophagy Modulators: Kim et al. [7] explored parallels between SCD and Granular Corneal Dystrophy type 2, proposing treatments targeting oxidative damage and autophagy, such as rapamycin and melatonin. These approaches could provide novel avenues for managing SCD in the future, although clinical trials are essential for validation. Nanotechnology-based ocular drug delivery offers a promising approach for SCD management. Gold nano-urchins significantly enhance corneal drug retention, improving tear production, reducing IL-6 expression, suppressing angiogenesis, and promoting nerve regeneration in preclinical models [19]. These innovations may help address oxidative stress, inflammation, and neurodegeneration in SCD.
- Gene Therapy: With the identification of UBIAD1 mutations as the primary genetic driver of SCD, gene therapy targeting cholesterol metabolism pathways is a promising yet undeveloped frontier. Advances in CRISPR-based gene editing may enable future correction of pathogenic mutations, offering a potential cure.
- Lipid-Lowering Agents: Systemic lipid-lowering medications, such as ezetimibe or fibrates, may be explored in select patients with concomitant hyperlipidemia, although their role in preventing corneal lipid accumulation remains uncertain.

For our patient, continued follow-up is necessary to monitor her disease progression and evaluate emerging therapeutic options. Future research should focus on refining these interventions and assessing their long-term efficacy in clinical settings.

### 3.6. Implications for Multidisciplinary Care

This case underscores the importance of a multidisciplinary approach in managing SCD. Collaboration between ophthalmologists, geneticists, and systemic specialists, such as rheumatologists and cardiologists, is critical for addressing the complex interplay between ocular and systemic health. Future research should aim to better elucidate the relationship between systemic diseases, such as rheumatoid arthritis, and SCD, as well as explore novel therapeutic strategies targeting the disease’s molecular and genetic basis.

Establishing a registry for Schnyder corneal dystrophy and other rare ocular disorders would be valuable in offering information about the disease course, genotype–phenotype correlations, and outcomes after treatments. National rare disease registries in France, the United States, and Nordic nations are established and are used for research, trial enrollment, and guideline generation. A European or global SCD registry would enhance awareness and direct the generation of guidelines based on evidence for treating SCD.

## 4. Conclusions

This case of Schnyder corneal dystrophy highlights the complex interplay between ocular and systemic health in rare corneal disorders. The patient’s presentation, characterized by bilateral stromal deposits, photophobia, and systemic comorbidities, underscores the diagnostic and management challenges associated with SCD. While advances in understanding the genetic and molecular basis of the disease, particularly the role of UBIAD1 mutations, have provided valuable insights, the absence of universally accessible genetic testing and limited therapeutic options remain significant barriers to optimal care.

This case reinforces the importance of a multidisciplinary approach, integrating ophthalmological, systemic, and genetic evaluations to address the full spectrum of SCD manifestations. Emerging therapeutic strategies, such as targeting oxidative stress and vitamin K metabolism, offer promise but require further research and clinical validation. Future studies should focus on expanding diagnostic tools, exploring genotype–phenotype correlations, and developing non-invasive treatments to improve outcomes for patients with SCD. This case contributes to the growing body of research, emphasizing the need for individualized and collaborative care in managing this rare disorder.

## Data Availability

Data are contained within the article.
